# Handling missing MRI sequences in deep learning segmentation of brain metastases: a multicenter study

**DOI:** 10.1038/s41746-021-00398-4

**Published:** 2021-02-22

**Authors:** Endre Grøvik, Darvin Yi, Michael Iv, Elizabeth Tong, Line Brennhaug Nilsen, Anna Latysheva, Cathrine Saxhaug, Kari Dolven Jacobsen, Åslaug Helland, Kyrre Eeg Emblem, Daniel L. Rubin, Greg Zaharchuk

**Affiliations:** 1 Department of Diagnostic Physics, Oslo University Hospital, Oslo, Norway; 2 Department of Radiology, Stanford University, Stanford, USA; 3 Faculty of Health and Social Sciences, University of South-Eastern Norway, Drammen, Norway; 4 Department of Biomedical Data Science, Stanford University, Stanford, USA; 5 Department of Radiology and Nuclear Medicine, Oslo University Hospital, Oslo, Norway; 6 Department of Oncology, Oslo University Hospital, Oslo, Norway

**Keywords:** CNS cancer, Learning algorithms, Machine learning

## Abstract

The purpose of this study was to assess the clinical value of a deep learning (DL) model for automatic detection and segmentation of brain metastases, in which a neural network is trained on four distinct MRI sequences using an input-level dropout layer, thus simulating the scenario of missing MRI sequences by training on the full set and all possible subsets of the input data. This retrospective, multicenter study, evaluated 165 patients with brain metastases. The proposed input-level dropout (ILD) model was trained on multisequence MRI from 100 patients and validated/tested on 10/55 patients, in which the test set was missing one of the four MRI sequences used for training. The segmentation results were compared with the performance of a state-of-the-art DeepLab V3 model. The MR sequences in the training set included pre-gadolinium and post-gadolinium (Gd) T1-weighted 3D fast spin echo, post-Gd T1-weighted inversion recovery (IR) prepped fast spoiled gradient echo, and 3D fluid attenuated inversion recovery (FLAIR), whereas the test set did not include the IR prepped image-series. The ground truth segmentations were established by experienced neuroradiologists. The results were evaluated using precision, recall, Intersection over union (IoU)-score and Dice score, and receiver operating characteristics (ROC) curve statistics, while the Wilcoxon rank sum test was used to compare the performance of the two neural networks. The area under the ROC curve (AUC), averaged across all test cases, was 0.989 ± 0.029 for the ILD-model and 0.989 ± 0.023 for the DeepLab V3 model (*p* = 0.62). The ILD-model showed a significantly higher Dice score (0.795 ± 0.104 vs. 0.774 ± 0.104, *p* = 0.017), and IoU-score (0.561 ± 0.225 vs. 0.492 ± 0.186, *p* < 0.001) compared to the DeepLab V3 model, and a significantly lower average false positive rate of 3.6/patient vs. 7.0/patient (*p* < 0.001) using a 10 mm^3^ lesion-size limit. The ILD-model, trained on all possible combinations of four MRI sequences, may facilitate accurate detection and segmentation of brain metastases on a multicenter basis, even when the test cohort is missing input MRI sequences.

## Introduction

Advances in artificial intelligence (AI) are suggesting the possibility of new paradigms in healthcare and are particularly well-suited to be adopted by radiologists^1–4^. In recent years, there has been significant effort in utilizing the next-generation AI technology, coined deep learning, to learn from labeled magnetic resonance imaging (MRI) data^5–7^. One key advantage of AI-based radiology is the automatization and standardization of tedious and time-consuming tasks, most clearly exemplified in the tasks surrounding detection and segmentation^8–10^. Several deep learning approaches have successfully been developed and tested for automatic segmentation of gliomas^11–13^, thanks in part to the publicly available brain tumor segmentation (BraTS) dataset^14^. In recent years, studies have also shown the potential of AI-based segmentation in patient cohorts comprising tumor subtypes, such as brain metastases, which may pose a greater challenge in terms of segmentation performance given their wide range of sizes and multiplicity^15,16^. Delineation of initial metastatic lesion size and changes related to disease progression or response are key neuroradiology tasks^17^. Traditionally, the metrics used for assessing brain metastases are based on unidimensional measurements, and although the value of using volumetric measurements has been increasingly discussed, expert groups remain reluctant to endorse a universal requirement of volumetric criteria for assessing brain metastases. One concern often raised is that volumetric analysis, as performed manually by radiologist, adds cost and complexity, and is not available at all centers. Consequently, there has been a strong need to develop an accurate pipeline capable of automatic detection and segmentation of brain metastases. In a recent study, we trained a fully convolution neural network (CNN) for automatic detection and segmentation of brain metastases using multisequence MRI^18^. While our DL-approach showed high performance, the robustness and clinical utility needs to be challenged in order to fully understand its strengths and limitations. In fact, many AI-based segmentation studies are limited in terms of generalizability in that the algorithms are trained and tested on single-center patient cohorts. In some studies, the training-sets and test-sets are even limited to a single magnetic field strength, a single vendor, and/or a single scanner for data acquisition. A key step towards understanding the generalizability and clinical value of any deep neural network is by training and testing using real-world multicenter data. Another limitation of these AI-based segmentation networks is that they are trained on a distinct set of MRI contrasts, which limits the use of the networks to sites acquiring the same sequences. However, deep neural networks should be able to handle missing model inputs. To this end, this work tested an AI-based segmentation model, called input-level dropout (ILD), in which a neural network with an input dropout layer is trained on the full set of four distinct MRI sequences, as well as every possible subset of the MRI sequences. Hence, our proposed model can improve the generalizability of deep learning segmentation models by enabling inference at imaging sites missing MRI sequences used for training. This proposition was investigated by testing the trained ILD-model on a patient cohort acquired at a different site and missing one of the four MRI sequences used for training. To evaluate this network’s performance, a second neural network was trained and tested using state-of-the-art architecture without applying the input-level dropout strategy, i.e., only trained on the limited sequences corresponding to those in the test set. We hypothesize that the ILD-model will yield segmentation performance comparable to that of a state-of-the-art segmentation network, while at the same time being robust towards missing input data and allow it to generalize to multicenter MRI data.

## Results

### Training and inference time

The total time used for training was approximately 20 h for both the ILD-model and the DeepLab V3 network. For processing a test case using the ILD-model, the forward pass on a system with two NVIDIA GTX 1080Ti GPUs took approximately 250 ms per slice.

### Network performance

**Fig. 1:**
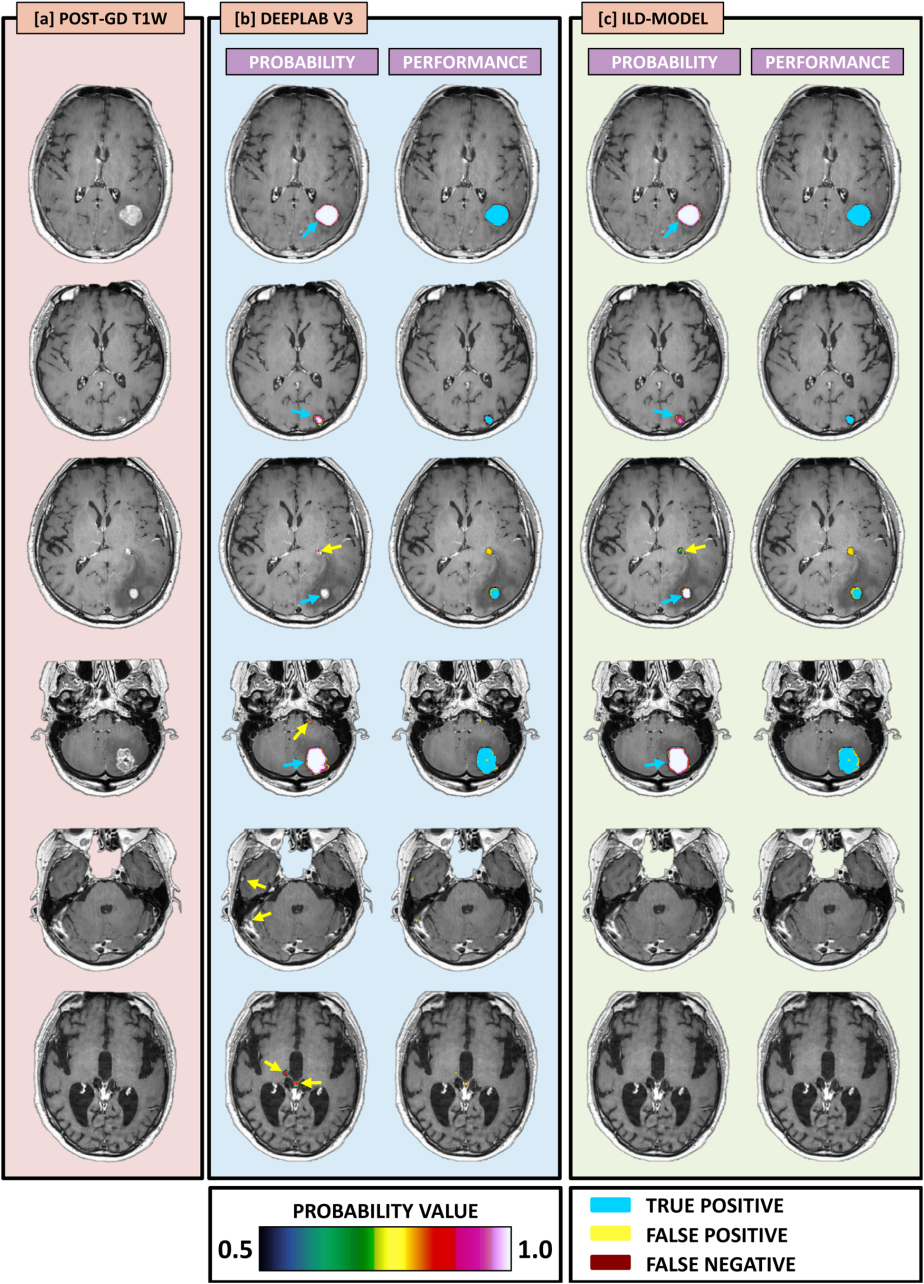
Example cases—Examples in representative test set cases showing the segmentation predictions from the DeepLab V3 network and the ILD-method. The image mosaic shows the post-Gd 3D T1-weighted image-series (**a**), and the predictions as probability maps (voxel-wise ranging from 0.5 to 1 as indicated by the color bar) and performance maps (classified as true negative, false positive, and false negative as specified by the color code) from the DeepLab V3 network (**b**) and the ILD-method (**c**). All maps are shown as overlays on a post-Gd 3D T1-weighted image-series. The cases shown here are [first row] a 65-year-old female with malignant melanoma, [second row], 73-year-old male with non-small cell lung cancer (NSCLC), [third row] 66-year-old male with NSCLC, [fourth row] 44-year-old female with NSCLC, [fifth row] 64-year-old female with NSCLC, and [sixth row] 73-year-old male with NSCLC. The blue arrows indicate true positive lesions, while yellow arrows indicate false positive lesions. Note that in the bottom three cases, the DeepLab V3 returns several false positive lesions which are not reported by the ILD-method, thus reflecting the results indicating a superior performance on false positive rate by the ILD-method.

**Fig. 2:**
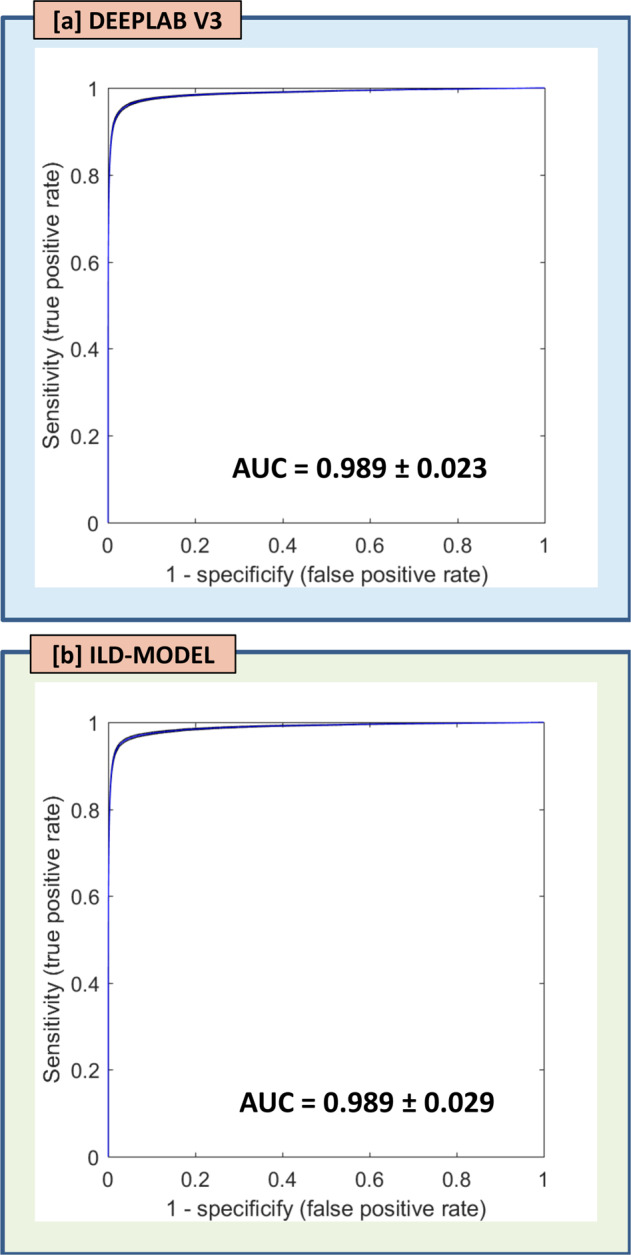
Voxel-wise detection accuracy. ROC curves with 95% confidence interval (shaded area) averaged across all 55 test cases for the (**a**) DeepLab V3 model and the (**b**) ILD-method. The area under the ROC curve was 0.989 (ranging from 0.896 to 1.000) for the DeepLab V3 model, and 0.989 (ranging from 0.845 to 1.000) for the ILD-model.

Figure 1 shows six example cases demonstrating the resulting probability maps, as well as maps representing the performance in terms of true positive, false positive, and false negative, as an overlay on the post-Gd 3D T1-weighted spin echo image-series. The ILD- and DeepLab V3 model performance are summarized in Table 1. Both the ILD- and DeepLab V3 models show a high voxel-wise detection accuracy, yielding an AUC, averaged across all test cases, of 0.989 ± 0.029 and 0.989 ± 0.029 (NS, *p* = 0.620), respectively (Fig. 2).

**Table 1:** Detection accuracy and segmentation performance.

Voxel-based statistics							Lesion-based statistics	
Network	AUC ROC	DICE	IoU	Recall	Precision	FPR	FP (no size limit)	FP (10 mm^3^ size limit)
DeepLab V3	0.989 ± 0.023	0.774 ± 0.104	0.492 ± 0.186	0.631 ± 0.208	0.722 ± 0.206	0.001 ± 0.001	26.3 ± 17.2	7.0 ± 5.3
ILD-model	0.989 ± 0.029	0.795 ± 0.105	0.561 ± 0.225	0.671 ± 0.262	0.790 ± 0.158	0.001 ± 0.001	12.3 ± 10.2	3.6 ± 4.1
*p*-value	0.620	0.017	<0.001	0.167	0.095	0.065	<0.001	<0.001

All metrics except AUC ROC were estimated using a probability threshold of 0.87 for the DeepLab V3 model, and 0.76 for the DropOut model.

**Fig. 3:**
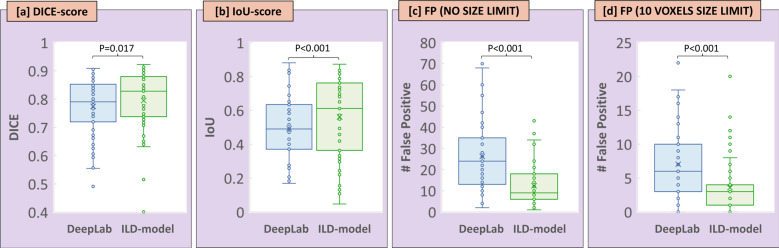
Segmentation performance. Segmentation performance Boxplot showing the resulting (**a)** Dice-score and (**b)** IoU-score, as well as the number of false positive (**c)** without and (**d)** with a lesion-size limit of 10 mm^3^, as determined from the segmentation probability maps produced by the DeepLab V3 model (blue) and the in-house ILD-method (green). Boxplots illustrate median (center line) and mean (X inside box) values, interquartile range (bounds of box), minimum and maximum values (whiskers), and outliers (circles outside whiskers).

Based on the ROC analysis on the validation set, an optimal probability threshold for including a voxel as a metastasis was set to 0.76 for the ILD-model, and 0.87 for the DeepLab V3 network. Using these thresholds, the ILD-model demonstrated a significantly higher Dice-score (0.795 ± 0.104 vs. 0.774 ± 0.104, *p* = 0.017), and IoU-score (0.561 ± 0.225 vs. 0.492 ± 0.186, *p* < 0.001), compared to the DeepLab V3 network (Fig. 3). The average recall and precision values were also higher for the ILD-model, but this difference was not statistically significant (Table 1).

On a per-lesion basis, and without any lesion-size limit, the ILD-model showed an average FP of 12.3/patient, which was significantly lower that the DeepLab V3 network (26.3/patient, *p* < 0.001). By applying a lesion-size limit of 10 mm^3^, the ILD-model demonstrated an average FP of 3.6/patient, also significantly lower that the DeepLab V3 (7.0/patient, *p* < 0.001) (Fig. 3).

## Discussion

Detection and segmentation of brain metastases on radiographic images sets the basis for clinical decision making and patient management. Precise segmentation is crucial for several steps in the clinical workflow such as treatment decision, radiation planning, and assessing treatment response, and must be performed with the utmost accuracy. Considering that the value of volumetric measurements of enhancing brain lesions are increasingly discussed^17^, future manual detection and segmentation pose a tedious and time-consuming challenge, particularly with the growing use of multisequence 3D imaging.

In this study, we demonstrated the clinical value of the ILD-model for automatic detection and segmentation of brain metastases. This neural network has a unique advantage over other segmentation networks because it uses an input dropout layer; trained on a full set of four MRI input sequences, as well as every possible reduced subset of the input channels, thus simulating the scenario of missing MRI sequence data. Consequently, the resulting ILD-model can return a model output (probability map) regardless of missing input MRI sequences. The accuracy of the ILD-model in detecting metastatic voxels in the brain, as measured by the AUC, is equivalent to that of the state-of-the-art DeepLab V3 neural network trained on the specific subset of sequences in our test set. However, our results indicate that the proposed model is superior to the DeepLab V3 in terms of segmentation performance, as measured by the Dice score and IoU, while at the same time returning significantly fewer FP. Nevertheless, the number of FP reported by the ILD-model remains a challenge that needs to be addressed^19^. These errors were typically seen in and near vascular structures. It is hypothesized that adding other MRI sequences in the training and test data, e.g., diffusion weighted MRI, may reduce the number of FP. Further, note that the improved generalization was achieved despite differences in the patient demographics between the training and test sets, with more frequent representation of lung and melanoma metastases in the test set. Finally, we would like to emphasize that the ILD-model does not require retraining for another subset of imaging sequences that might be acquired in another institution.

The neural networks used in this study were based on the DeepLab V3 architecture, which is considered as one of the most robust neural networks for image-based semantic segmentation. The key difference of the DeepLab V3 compared with other relevant networks is its reliance on atrous (or dilated) convolutions. By using atrous convolutional layers, our network has a large receptive field, thereby incorporating greater spatial context. This approach may be key to enabling the network to identify local features as well as global contexts, i.e., identifying brain regions, which could enhance the network’s decision-making process on similar local features.

In our study, the networks’ performance was tested on multicenter data, representing an essential step towards understanding the generalizability and clinical value of the proposed neural network. In this sense, it represents a logical extension of our prior single-center study on this topic^18^. No previous studies have evaluated the deep learning for brain metastasis detection using multicenter data. Other single-center studies, such as Liu et al.^15^ and Charron et al.^16^, have recently shown that deep neural networks can detect and segment brain metastases with high performance, reporting results comparable to that of the current study. The latter study also demonstrated that a deep neural network trained on multisequence MRI data outperformed single contrast networks.

In general, the two most used MRI sequences for assessing brain metastases are post-Gd T1-weighted and T2-weighted FLAIR. The post-Gd 3D T1-weighted, high-resolution isotropic sequence is most crucial^20^ and can be acquired by fast spin-echo or gradient-echo techniques. The 3D T1-weighted gradient-echo sequences (e.g., IR-FSPGR, BRAVO, and MPRAGE) are broadly used because they create isotropic T1-weighted images with excellent gray-white matter differentiation, but are limited by lower contrast conspicuity and a lack of blood vessel suppression. The 3D fast spin-echo techniques (e.g., CUBE, SPACE, and VISTA) are relatively newer techniques optimized for isotropic high-resolution 3D imaging of T1-weighted, T2-weighted, or FLAIR images, and have the advantage of blood vessel suppression. For this study, post-Gd T1-weighted 3D fast spin-echo, pre-Gd and post-Gd T1-weighted 3D axial IR-FSPGR, and 3D FLAIR sequences were used as input to train the neural network. While these sequences are widely used for imaging brain metastases, they are not compulsory. Variations in sequences and acquisition parameters among different institutions frequently are present. For instance, 2D FLAIR (with thicker slice and non-isotropic voxels) may be acquired instead of 3D FLAIR. In clinical practice, it is also not unusual to omit sequences owing to patients’ safety or comfort. Therefore, it is imperative to design a robust and versatile neural network that can accommodate missing sequences while maintaining good performance. To this end, the goal of this study was to develop a deep learning model that is able to detect and segment brain metastasis with high accuracy, equivalent to that of the state-of-the-art DeepLab V3 model, even when the clinical end-user does not have access to all MRI data on which the model was trained. This is a major improvement for the generalizability of deep learning segmentation tools since many clinical sites do not the time or hardware to perform all four MRI scans.

While this study shows a high accuracy using the ILD-model for detecting and segmenting brain metastases, the results should be interpreted in light of the limited sample size and the homogeneity of the test cohort. Patients included in the test set were all scheduled for SRS, which generally presents with fewer and larger metastases, which in turn may be easier for the network to predict. This hypothesis is supported by observations made in our previous study, in which the tested neural network showed higher accuracy in patients with three or less metastases compared to patients with >3 metastases^18^. However, a total of nine patients in the current test set presented with >3 small metastases, for which the ILD-model still demonstrated a high accuracy and performance, equivalent to the average metrics for all lesions.

In conclusion, this study demonstrates that the ILD-model, utilizing a pulse sequence level dropout layer, thus being trained on all possible combinations of multiple MRI sequences, can detect and segment brain metastases with high accuracy, even when the test cohort is missing MRI data. This is likely of value for generalizing deep learning models for use in multiple different imaging sites.

## Methods

### Patient population

This retrospective, multicenter study was approved by the Oslo University Hospital and Stanford Review Board. The patient cohort consisted of a total of 165 patients with brain metastases, enrolled from two different hospitals, hereinafter referred to as “Hospital A” and “Hospital B”. From Hospital A, MRI data from a total of 100 patients were acquired and used for neural network training. These patients received their scans for clinical purposes, and our Review Board waived the requirement for informed consent. Further, a total of 65 patients from Hospital B were used for validation and testing, and written informed consent was obtained from all the patients.

Inclusion criteria for the training data included the presence of known or possible metastatic disease (i.e., presence of a primary tumor), no prior surgical or radiation therapy, and the availability of all required MR imaging sequences (see below). Only patients with ≥1 metastatic lesion were included. Mild patient motion was not an exclusion criterion. For the validation and test data, we used MRI data from an ongoing clinical study (NCT03458455) conducted at Hospital B. To be eligible for inclusion, patients had to receive stereotactic radiosurgery (SRS) for at least one brain metastasis measured at a minimum of 5 mm in one direction, be untreated or progressive after systemic or local therapy, have confirmed non-small-cell lung cancer (NSCLC) or malignant melanoma, be ≥18 years of age; have an Eastern Cooperative Oncology Group performance status score ≤1, and have a life expectancy >6 weeks. Details on the patient cohorts are shown in Table 2.

**Table 2:** Patient demographics.

	Hospital A	Hospital B
# of patients	100	65
Gender	71 F/29 M	35 F/30 M
Mean age (range)	64 (32–92)	65 (32–86)
*Primary cancer:*		
Lung	66	45
Skin/melanoma	4	20
Breast	26	–
Genitourinary	2	–
Gastrointestinal	2	–

### Imaging protocol

MRI training data was acquired on both 1.5 T (*n* = 7; TwinSpeed and SIGNA Explorer, GE Healthcare, Chicago, USA) and 3 T (*n* = 93; SIGNA Architect, and Discovery 750 and 750w, GE Healthcare, Chicago, USA; Skyra, Siemens Healthineers, Erlangen, Germany) scanners. The training set included four MRI sequences; post-Gd T1-weighted 3D axial inversion recovery prepped fast spoiled gradient-echo (IR-FSPGR) (BRAVO/MPRAGE), pre- and post-Gd T1-weighted 3D fast spin echo (CUBE/SPACE), and 3D CUBE/SPACE fluid-attenuated inversion recovery (FLAIR). A dose of 0.1 mmol/kg body weight of gadobenate dimeglumine (MultiHance, Bracco Diagnostics, Princeton, USA) was intravenously injected for Gd-enhancement. For the test set (*n* = 65), imaging was performed on a 3 T Skyra scanner (Siemens Healthineers, Erlangen, Germany), and included three MRI sequences; pre-Gd and post-Gd T1-weighted 3D fast spin echo (SPACE) and 3D T2-weighted FLAIR. Note that the 3D T1 BRAVO sequence is missing from the test set. All sequences with key imaging parameters are summarized in Table 3.

**Table 3:** Overview of MRI pulse sequences and key imaging parameters.

Technique	3D T1 BRAVO	Pre/Post 3D T1 CUBE/SPACE	3D FLAIR
*Hospital A data*			
TR (ms)^a^	12.02/8.24	550/602	6000
TE (ms)^a^	5.05/3.24	9.54/12.72	119/136
Flip angle^a^	20/13	90	90
FOV (mm2)	240 × 240	250 × 250	240 × 240
Inversion time (ms)^a^	300/400	–	1880/1700
Acquisition matrix	256 × 256	256 × 256	256 × 256
Slice thickness (mm)	1	1	1–1.6
# of slices	160	270–320	270–320
Slice acquisition plane	Axial	Sagittal	Sagittal
*Hospital B data*^b^			
TR (ms)	–	700	5000
TE (ms)	–	12	387
Flip angle	–	120	120
FOV (mm^2^)	–	230 × 230	230 × 230
Inversion time (ms)	–	–	1800
Acquisition matrix	–	256 × 256	256 × 256
Slice thickness (mm)	–	0.9	0.9
# of slices	–	192	208
Slice acquisition plane	–	Sagittal	Sagittal

*TR* repetition time, TE echo time, FOV field-of-view, BRAVO-T1-weighted inversion recovery prepped fast spoiled gradient-echo, CUBE/SPACE-T1-weighted fast spin-echo, FLAIR fluid attenuated inversion recovery.

^a^In case of varying parametric values between field strength,“/” notation is given (1.5 T/3 T).

^b^Note that the Hospital B data is missing 3D T1 BRAVO images.

### Image pre-processing and segmentation

For the training data, ground truth segmentations for every enhancing metastatic lesion were determined by two neuroradiologists. Specifically, a neuroradiologist with 3 years of experience manually delineated each enhancing brain lesion by placing a region of interest (ROI) over each image slice where a lesion was visible on the Gd-enhanced IR-FSPGR sequence. The combined ROIs for a specific lesion comprised the volume of interest (VOI). In a separate session one week later, the second neuroradiologist with 9 years of experience confirmed (and modified as appropriate) final VOI placement for each lesion. All lesions were outlined using the OsiriX MD software package (Version 8.0, Geneva, Switzerland).

For the test data, ground truth segmentations of Gd-enhancing metastatic lesions were manually drawn on post-Gd 3D T1-weighted spin echo data by two radiologists with 14 and 5 years of relevant experience. Delineations were performed using the nordicICE software package (NordicNeuroLab, Bergen, Norway).

All image-series were co-registered to a common anatomical space. For the training data, pre-Gd and post-Gd 3D T1-weighted spin echo data and FLAIR were co-registered to the post-Gd 3D T1-weighted IR-FSPGR, whereas for the test data, the post-Gd 3D T1-weighted spin echo images was used as reference for the pre-Gd 3D T1-weighted spin echo data and FLAIR. Prior to network training, a defacing procedure was applied to anonymize all imaging data using an in-house algorithm (MATLAB R2017a version 9.2.0, MathWorks Inc. Natick, MA).

### Neural network details

**Fig. 4:**
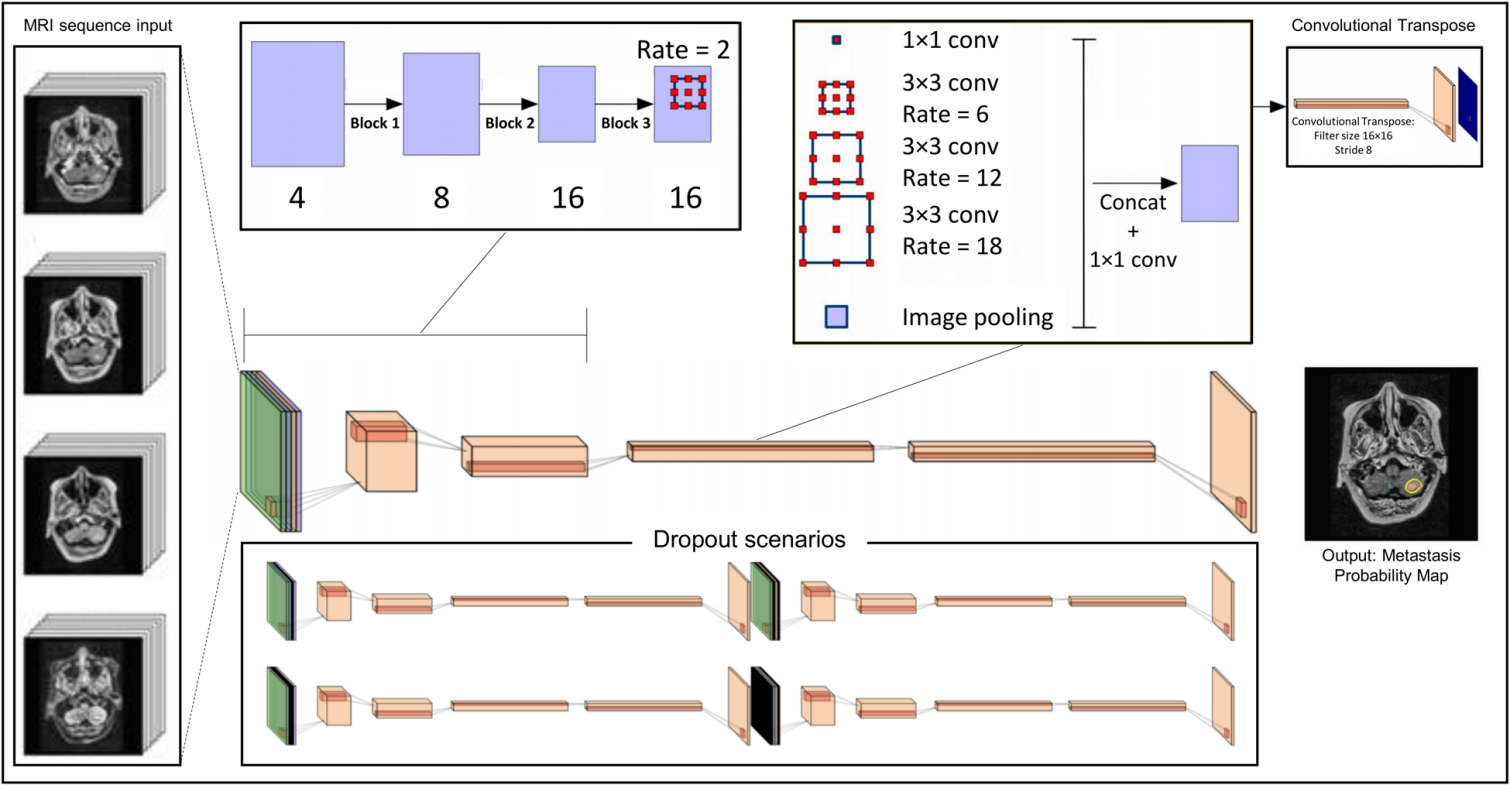
Neural network architecture—Diagram showing the ILD-model architecture used in this study. Five contiguous axial slices of each of the four pulse sequences (BRAVO, pre-Gd and post-Gd CUBE, and T2-weighted FLAIR) are concatenated in the color-channel dimension to create an input tensor with channel dimension 20. This is fed into the neural network to predict the segmentation on the center slice. The pipeline is built largely on the DeepLab V3 architecture^21^.

The neural networks used in this study were based on the DeepLab V3 architecture^21^, and the modifications and training strategies are detailed in a recent work^22^. This study utilized and trained a “input-level integration dropout” network, referred to as the ILD-model (Fig. 4). This model was trained on patients from Hospital A, using five slices from the four aforementioned pulse sequences as input. These were all stacked together in the color channel, resulting in an image tensor of shape 256 × 256 × 20 as the model input. The network was trained by utilizing a pulse-sequence level dropout^23,24^, replacing the full five slices of any given pulse sequence with an empty tensor of 0’s during training; thus enabling the network to handle missing MRI pulse sequence input during inference. This yields a network trained on a single data center with a superset of pulse sequences to what may be used in practice. The network was trained using PyTorch, and the resulting output was a probability map of whether the voxel represents a metastasis, ranging from 0 to 1.

With the ILD-model, we propose stochastically zeroing out random MRI sequences during training. By training on inputs with missing MRI sequences, this model should be robust to similar scenarios during inference. With this setup, it is important to consider the weighting function. Dropping out neurons with a probability, *p*, during training, but having them active during inference creates a difference in the sum of neuron activations. This can be solved by eighter upweighting the dropout-layer neurons by a factor of 1/1 − *p* during training or downweighting the same neurons by a factor of 1 − *p* during inference. In this work, the input dropout layer was upweighted by a factor of 1/1 − *p*, where *p* is the proportion of dropped out MRI sequences, in both training and inference. It is important to note that dropout cannot solely be done on a statistical basis. Normally, each channel has a certain probability of being dropped. However, given our 2.5D network structure, eighter all or none of the *z*-slices of a certain MRI sequence must be dropped out. In addition, all four MRI sequences must never drop out during training. This will result in a model receiving an input tensor of all 0 s, which would be intractable and lead to unstable training. Figure 4 show the standard input-level integration architecture and the four potential “dropped out” versions. From our four input-concatenated pulse sequences, we randomly drop out 0–3 input channels (shown in black).

To evaluate the segmentation performance of the ILD-model, a second neural network was trained and tested using the state-of-the-art DeepLab V3 architecture without applying the input-level dropout strategy, and only trained on the complete set of sequences matching that of the test set from Hospital B (i.e., excluding the post-Gd 3D T1-weighted IR-FSPGR sequence).

All patients from Hospital A were used for training, while the patients from Hospital B were divided into validation-sets and test-sets, giving a final breakdown of 100 training cases, 10 validation cases, and 55 test cases. All training was done on a system with two NVIDIA GTX 1080Ti GPUs.

### Statistical analysis

In this study, we perform statistical analysis on a voxel-by-voxel and lesion-by-lesion basis. The voxel-based approach allows investigation of differences in the probability maps at the smallest volume-level of the MR image-series. ROC curve statistics was used to evaluate the networks’ ability to differentiate between healthy and metastatic tissue on a voxel-by-voxel basis. For each patient in the test set, the area under the ROC curve (AUC) was measured. Further, the optimal probability threshold for including a voxel within the metastatic lesion was determined using the Youden index from the ROC statistics on the validation set. Using this threshold, the networks segmentation performance was further evaluated by estimating the precision-values and recall-values, false positive rate (FPR), as well as the IoU and Dice similarity score. The networks’ performance was also evaluated on a per-lesion basis by calculating the number of false positive (FP) per case. This metric was determined by multiplying the ground truth maps and the thresholded probability maps and counting the number of overlapping objects in the resulting binary image. By using a connecting component approach, voxels were considered connected if their edges or corner touch. The number of FP was determined both without any size criterion, as well as only considering objects ≥10 mm^3^ (roughly 2 mm in linear dimension) as a detected lesion.

Finally, the performance of the ILD-model and the DeepLab V3 network was compared using the Wilcoxon rank sum test. A *p*-value of 5% or lower was considered to be statistically significant. All Statistical analyses were performed using MATLAB R2017a version 9.2.0 (MathWorks Inc. Natick, MA).

### Reporting summary

Further information on research design is available in the Nature Research Reporting Summary linked to this article.

## Supplementary information

Reporting Summary

## Data Availability

The data are available upon reasonable request.

## Code availability

The code is available upon reasonable request.
