# Supplementary material for: Handling missing MRI sequences in deep learning segmentation of brain metastases: a multicenter study
Source: NPJ Digit Med. 2021 Feb 22;4:33. doi: 10.1038/s41746-021-00398-4 (PMC7900111; doi:10.1038/s41746-021-00398-4)
Supplement: Supplementary file 1 — Reporting Summary [file 41746_2021_398_MOESM1_ESM.pdf]

nature research

Corresponding author(s): Greg Zaharchuk

Last updated by author(s): 13/12/20

Reporting Summary

Nature Research wishes to improve the reproducibility of the work that we publish. This form provides structure for consistency and transparency in reporting. For further information on Nature Research policies, see our Editorial Policies and the Editorial Policy Checklist.

Statistics

For all statistical analyses, confirm that the following items are present in the figure legend, table legend, main text, or Methods section.

n/a	Confirmed	
☐	☒	The exact sample size (*n*) for each experimental group/condition, given as a discrete number and unit of measurement
☐	☒	A statement on whether measurements were taken from distinct samples or whether the same sample was measured repeatedly
☐	☒	The statistical test(s) used AND whether they are one- or two-sided<br>*Only common tests should be described solely by name; describe more complex techniques in the Methods section.*
☐	☒	A description of all covariates tested
☐	☒	A description of any assumptions or corrections, such as tests of normality and adjustment for multiple comparisons
☐	☒	A full description of the statistical parameters including central tendency (e.g. means) or other basic estimates (e.g. regression coefficient) AND variation (e.g. standard deviation) or associated estimates of uncertainty (e.g. confidence intervals)
☐	☒	For null hypothesis testing, the test statistic (e.g. *F*, *t*, *r*) with confidence intervals, effect sizes, degrees of freedom and *P* value noted<br>*Give P values as exact values whenever suitable.*
☒	☐	For Bayesian analysis, information on the choice of priors and Markov chain Monte Carlo settings
☒	☐	For hierarchical and complex designs, identification of the appropriate level for tests and full reporting of outcomes
☒	☐	Estimates of effect sizes (e.g. Cohen's *d*, Pearson's *r*), indicating how they were calculated

Our web collection on statistics for biologists contains articles on many of the points above.

Software and code

Policy information about availability of computer code

Data collection	Conventional user interface for GE and Siemens scanners
Data analysis	Manual delineations of lesions in data from Hospital a were performed using the OsiriX MD software package (Version 8.0, Geneva, Switzerland).<br><br>Manual delineations of the lesions in the test set were performed using the nordicICE software package (NordicNeuroLab, Bergen, Norway). No specific version.<br><br>A defacing procedure was applied to anonymize all imaging data using an in-house algorithm (MATLAB R2017a version 9.2.0, MathWorks Inc. Natick, MA).

For manuscripts utilizing custom algorithms or software that are central to the research but not yet described in published literature, software must be made available to editors and reviewers. We strongly encourage code deposition in a community repository (e.g. GitHub). See the Nature Research guidelines for submitting code & software for further information.

Data

Policy information about availability of data

All manuscripts must include a data availability statement. This statement should provide the following information, where applicable:

- Accession codes, unique identifiers, or web links for publicly available datasets
- A list of figures that have associated raw data
- A description of any restrictions on data availability

The data are available upon reasonable request.

Field-specific reporting

Please select the one below that is the best fit for your research. If you are not sure, read the appropriate sections before making your selection.

☒ Life sciences ☐ Behavioural & social sciences ☐ Ecological, evolutionary & environmental sciences

For a reference copy of the document with all sections, see nature.com/documents/nr-reporting-summary-flat.pdf

Life sciences study design

All studies must disclose on these points even when the disclosure is negative.

Sample size	No sample-size calcualtions was performed. This retrospective, multi-center study included patient cohorts consisted of a total of 165 patients with brain metastases, enrolled from two different hospitals, hereinafter referred to as 'Hospital A' and 'Hospital B'. From Hospital A, a total of 100 patients. These patients received their MRI scans for clinical purposes. Further, a total of 65 patients from Hospital B were included.
Data exclusions	Inclusion criteria for the data included from Hospital A was the presence of known or possible metastatic disease (i.e., presence of a primary tumor), no prior surgical or radiation therapy, and the availability of all required MR imaging sequences (see below). Only patients with ≥ 1 metastatic lesion were included. Mild patient motion was not an exclusion criterion. For the data from Hospital B, we used MRI data from an ongoing clinical study. To be eligible for inclusion, patients had to receive stereotactic radiosurgery (SRS) for at least one brain metastasis measured at a minimum of 5 mm in one direction, be untreated or progressive after systemic or local therapy, have confirmed non-small-cell lung cancer (NSCLC) or malignant melanoma, be ≥18 years of age; have an Eastern Cooperative Oncology Group performance status score ≤1, and have a life expectancy >6 weeks. Details on the patient cohorts are provided in the manuscript.
Replication	Concerning reproducibility, the segmentation performance of our proposed neural network was tested on multi-center data. Also, we are in the process of making our data available for the public, thus facilitating reproducibility assessments.
Randomization	The cases used for training the deep neural networks tested in this study were randomly selected from a clinical brain metastasis cohort.
Blinding	Blinding was not relevant for this study. Investigators were presented with relevant information and data in order to provide the most accurate annotation used as the ground truth for neural network training and testing.

Reporting for specific materials, systems and methods

We require information from authors about some types of materials, experimental systems and methods used in many studies. Here, indicate whether each material, system or method listed is relevant to your study. If you are not sure if a list item applies to your research, read the appropriate section before selecting a response.

Materials & experimental systems

n/a	Involved in the study	
☒	☐	Antibodies
☒	☐	Eukaryotic cell lines
☒	☐	Palaeontology and archaeology
☒	☐	Animals and other organisms
☐	☒	Human research participants
☐	☒	Clinical data
☒	☐	Dual use research of concern

Methods

n/a	Involved in the study	
☒	☐	ChIP-seq
☒	☐	Flow cytometry
☐	☒	MRI-based neuroimaging

Human research participants

Policy information about studies involving human research participants

Population characteristics	Patients from Hospital A: n=100; 71 female, 21 male; Mean age (range) = 64 (32 -92), Primary cancer: lung (n=66), skin (n=4), breast (n=26), genitourinary (n=2), gastrointestinal (n=2).<br><br>Patients from Hospital B: n=65; 35 female, 30 male; Mean age (range) = 65 (32 -86), Primary cancer: lung (n=45), skin (n=20).
Recruitment	Patients from Hospital A were recruited from a cohort receiving their scans for clinical purposes, while patients from Hospital B were recruited from an ongoing clinical study. To be eligible for inclusion at Hospital B, patients had to receive stereotactic radiosurgery (SRS) for at least one brain metastasis measured at a minimum of 5 mm in one direction, be untreated or progressive after systemic or local therapy, have confirmed non-small-cell lung cancer (NSCLC) or malignant melanoma, be ≥18 years of age; have an Eastern Cooperative Oncology Group performance status score ≤1, and have a life expectancy >6 weeks.
Ethics oversight	This study was approved by the Oslo University Hospital and Stanford Review Board.

Note that full information on the approval of the study protocol must also be provided in the manuscript.

Clinical data

Policy information about clinical studies

All manuscripts should comply with the ICMJE guidelines for publication of clinical research and a completed CONSORT checklist must be included with all submissions.

Clinical trial registration: NCT03458455

Study protocol: https://clinicaltrials.gov/ct2/show/NCT03458455

Data collection: Patients from Hospital A was enrolled in a clinical setting between June 2016 and June 2018 at Stanford University, Palo Alto, California, USA.

Patients from Hospital B was enrolled as part of a clinical study. Imaging was performed between January 2016 and December 2018 at Oslo University Hospital, Oslo, Norway.

Outcomes: The outcomes were defined as the neural networks ability to detect and segment brain metastases on multi-center data, and how the neural network handles missing MRI sequence input during inference.

These outcomes were assessed by utilizing expert annotations and using ROC-curve statistics, precision- and recall-values, false positive rate, as well as the Intersection over Union (IoU) and Dice similarity score.

Magnetic resonance imaging

Experimental design

Design type: Only conventional structural MR image-series with different contrast was used in this study (see below). No fMRI were performed.

Design specifications: Only 3D high spatial resolution MRI were performed. No fMRI data was acquired.

Behavioral performance measures: Not applicable

Acquisition

Imaging type(s): Structural.

Field strength: 1.5T and 3T

Sequence & imaging parameters: MRI data from Hospital A was acquired on both 1.5T (n=7; GE TwinSpeed and SIGNA Explorer) and 3T (GE SIGNA Architect, and Discovery 750 and 750w) scanners. The imaging protocol included post-Gadolinium (Gd) T1-weighted 3D axial inversion recovery prepped fast spoiled gradient- echo (IR-FSPGR) (BRAVO/MPRAGE), pre- and post-Gd T1-weighted 3D fast spin echo (CUBE/SPACE), and 3D CUBE/SPACE fluid-attenuated inversion recovery (FLAIR). A dose of 0.1 mmol/kg body weight of gadobenate dimeglumine (MultiHance, Bracco Diagnostics, Princeton, USA) was intravenously injected for Gd-enhancement. MRI data from Hospital B was performed on a 3T (n=65) Siemens Skyra scanner. The imaging protocol included pre- and post-Gd T1-weighted 3D fast spin echo (SPACE) and 3D T2-weighted FLAIR. Key imaging parameters are summarized below:

Hospital A:
- IR-FSPGR (1.5T/3T): TR=12.02/8.24ms, TE=5.05/3.24ms, FA=20/13, FOV=240x240mm2, TI=300ms/400ms, Acq.matrix=256x256, slice thickness=1mm.
- Pre-/post CUBE/SPACE (1.5T/3T): TR=550/602ms, TE=9.54/12.72ms, FA=90, FOV=250x250mm2, Acq.matrix=256x256, slice thickness=1mm
- FLAIR (1.5T/3T): TR=6000ms, TE=119/136ms, FA=90, FOV=240x240mm2, TI=1880/1700ms, Acq.matrix=256x256, slice thickness=1-1.6mm

Hospital B:
- Pre-/post SPACE: TR=700ms, TE=12ms, FA=120, FOV=230x230mm2, Acq.matrix=256x256, slice thickness=0.9mm
- FLAIR: TR=5000ms, TE=387ms, FA=120, FOV=230x230mm2, TI=1800ms, Acq.matrix=256x256, slice thickness=0.9

Area of acquisition: Conventional whole brain imaging.

Diffusion MRI: ☐ Used ☒ Not used

Preprocessing

Preprocessing software: Manual delineations of lesions in data from Hospital a were performed using the OsiriX MD software package (Version 8.0, Geneva, Switzerland).

Manual delineations of lesions in data from Hospital B were performed using the nordicICE software package (NordicNeuroLab, Bergen, Norway, no specific version).

	A defacing procedure was applied on all patients to anonymize all imaging data using an in-house algorithm (MATLAB R2017a version 9.2.0, MathWorks Inc. Natick, MA).
Normalization	Prior to neural network training, normalization was performed with independent histogram equalization on each slice of the 28-channel input.
Normalization template	All image-series were co-registered to a common anatomical space. For the data from Hospital A, pre- and post-Gd 3D T1-weighted spin echo data and FLAIR were co-registered to the post-Gd 3D T1-weighted IR-FSPGR, whereas for the data from Hospital B, the post-Gd 3D T1-weighted spin echo images was used as reference for the pre-Gd 3D T1-weighted spin echo data and FLAIR. No normalization template (e.i. MNI template) was used.
Noise and artifact removal	No procedure for artifact and structured noise removal was used.
Volume censoring	Not applicable

Statistical modeling & inference

Model type and settings	Receiver operating characteristic (ROC) curve statistics was used to evaluate the neural networks' ability to differentiate between healthy and metastatic tissue on a voxel-by-voxel basis. For each patient in the test set, the area under the ROC curve (AUC) was measured. Further, the optimal probability threshold for including a voxel within the metastatic lesion was determined using the Youden index from the ROC statistics on a validation set.
Effect(s) tested	The networks segmentation performance was evaluated by estimating the precision- and recall-values, false positive rate (FPR), as well as the Intersection over Union (IoU) and Dice similarity score. The networks' performance was also evaluated on a per-lesion basis by calculating the number of false positive (FP) per case.

Specify type of analysis: ☐ Whole brain ☐ ROI-based ☒ Both

Anatomical location(s): Brain

Statistic type for inference (See Eklund et al. 2016)	In this study, we perform statistical analysis on a voxel-by-voxel and lesion-by-lesion basis.
Correction	Not applicable

Models & analysis

n/a	Involved in the study
☒	☐ Functional and/or effective connectivity
☒	☐ Graph analysis
☐	☒ Multivariate modeling or predictive analysis

Multivariate modeling and predictive analysis	The deep neural network used in this study were based on a modified DeepLab V3 architecture with an input-level integration dropout strategy.
